# Isolated adrenocorticotropic hormone deficiency following immune checkpoint inhibitors treatment often occurs in polyglandular endocrinopathies

**DOI:** 10.1186/s12902-023-01397-0

**Published:** 2023-07-06

**Authors:** Hong Chen, Lei Zhang, Lin Zhao, Xiaomu Li

**Affiliations:** Department of Endocrinology and Metabolism, Zhongshan Hospital, Fudan University, No.180 Fenglin Road, Shanghai, 200032 China

**Keywords:** Immune checkpoint inhibitors, Isolated adrenocorticotropic hormone deficiency, Primary hypothyroidism, Fulminant type 1 diabetes mellitus

## Abstract

**Background:**

With the increasing application of immune checkpoint inhibitors (ICI) in cancer therapy, the occurrence of isolated adrenocorticotropic hormone deficiency (IAD), as an adverse effect, is also on the rise. Nevertheless, there are only a few studies regarding IAD induced by ICI. This study aimed at investigating the characteristics of IAD induced by ICI and its relationship with other endocrine adverse events.

**Methods:**

A retrospective study was conducted in the Endocrinology Department from January 2019 to August 2022 to investigate characteristics of patients with IAD. Clinical features, laboratory findings and treatment information were collected. All patients underwent a follow-up of 3-6-month.

**Results:**

28 patients with IAD were enrolled. All patients received treatment with anti-PD-1/ PD-L1. The median occurrence time of IAD was 24 (18–39) weeks after initiation of ICI treatment. Over half of the patients (53.5%) had an additional endocrinopathy, including primary hypothyroidism and fulminant type 1 diabetes mellitus (FT1DM), while other types of endocrinopathies were not identified. The interval between the occurrences of two gland damages was between 4 and 21 weeks or simultaneous. Primary hypothyroidism (46.4%) was more prevalent than FT1DM (7.1%). Fatigue and nausea were common symptoms, with a frequent occurrence of hyponatremia. All patients continued on oral glucocorticoids during follow-up.

**Conclusions:**

IAD induced by ICI could manifest independently, or more frequently in combination with hypothyroidism or FT1DM. This damage could happen at any point of ICI treatment. Given that IAD can be life-threatening, it is critical to evaluate pituitary function dynamically in patients undergoing immunotherapy.

## Background

Immune checkpoint inhibitors (ICI) are monoclonal antibodies for advanced tumor therapy by blocking cytotoxic-T cell lymphocyte antigen-4 (CTLA4) or programmed cell death receptor-1 (PD-1) and its ligand (PD-L1) [1]. However, despite their anti-tumor effects, ICI can result in unpredictable immune-related adverse events (irAEs), including various endocrine disorders through overactivated immune cells [2]. While some studies have suggested endocrine irAEs predict positive prognosis for certain types of cancer [3–5], some endocrine damages, such as adrenocorticotropic deficiency, can be fatal and often irreversible.

The incidence of isolated adrenocorticotropic hormone (ACTH) deficiency (IAD) has been growing due to the prevalence of ICI. IAD is characterized by secondary adrenal insufficiency with reduced or absent cortisol production, and normal secretion of pituitary hormones other than ACTH [6]. Sometimes, it can be challenging to recognize IAD, as its presentation is quite similar to those experienced during cancer therapy, such as fatigue, appetite loss, and weight loss [7, 8]. Furthermore, IAD may occur with other endocrine damages simultaneously or sequentially during ICI treatment or even after ICI cessation [9]. IAD may be overlooked when pituitary hormones are not measured routinely. Hence, it is essential to identify IAD patients and provide them with appropriate treatment.

To gain a better insight into IAD caused by ICI, we retrospectively reviewed patients with IAD induced by ICI in our department. We summarized their clinical features to improve our apprehension and awareness of this disorder, particularly from an endocrine perspective.

## Methods

### Participants and study design

This retrospective study was conducted at Department of Endocrinology of Zhongshan Hospital to investigate the characteristics of patients with ICI-induced IAD. Forty-four patients were consecutively referred to our department due to suspected or confirmed diagnosis of endocrine irAE from January 2019 to August 2022. All of them were Chinese. Information was extracted from electronic history record, including the type of cancer, the usage of ICI, clinical presentation, biochemical results, pituitary magnetic resonance imaging (MRI), and initial and follow-up treatment. All hormone and biochemistry measurements were performed at the central lab of Zhongshan hospital. Patients were followed up of 3–6 months after discharge from the Endocrinology Department. The exclusion criteria included: (1) secondary adrenal insufficiency combined with other pituitary hormone insufficiency; (2) endocrine gland disorders occurred prior to immunotherapy, including diabetes, hypothyroidism and adrenal insufficiency. All methods were carried out in accordance with the Declaration of Helsinki. This study was approved by the Ethics Committee of Zhongshan Hospital of Fudan university.

### Diagnostic criteria

Patients were evaluated when suspected of endocrine irAE, especially when they complained of fatigue, nausea and vomiting and headache, as well as with presence of hypotension or hyponatremia. Occurrence time to endocrinopathies was measured as the time to first documented hormone abnormality following receipt of the initial dose of ICI. For patients with decreased morning cortisol level, ACTH is used for differentiation between primary and secondary adrenal insufficiency [10]. Secondary adrenal insufficiency is defined as a morning cortisol value of < 100nmol/L (reference interval 133.0-537.0nmol/L) or a cortisol < 180 nmol/L in the context of critical stress, accompanied by normal or low ACTH (reference interval 1.5-13.9pmol/L) [11, 12]. IAD is defined as the presence of secondary adrenal insufficiency with normal secretion of the other pituitary hormones, as well as the absence of structural pituitary defects [6]. Although stimulation tests, such as the insulin -induced hypoglycemia test and ACTH stimulation test, are usually required to make a confirmation, patients in this study did not perform stimulation test due to their relatively severe symptoms. Pituitary MRI was also performed, when possible, to rule out other potential disorders particularly metastasis.

For better description, IAD was not classified into hypophysitis in our study. We defined ICI-related hypophysitis as the occurrence of two or more anterior pituitary hormone deficiencies, including ACTH (secondary adrenal insufficiency), TSH (secondary hypothyroidism), and follicle-stimulating hormone and luteinizing hormone (hypogonadotropic hypogonadism), regardless of whether posterior pituitary was affected [2]. Mild to moderate diffuse pituitary enlargement might be observed in pituitary MRI.

Overt primary hypothyroidism (PHT) was defined by a thyroid stimulating hormone (TSH) level above the upper reference limit (reference interval 0.27-4.2mIU/L), with a concomitant free thyroxine (FT4) level below the lower reference interval (reference interval 12.0-22.0pmol/L) or by TSH > 10.0mIU/L regardless of FT4. Subclinical hypothyroidism was defined as normal FT4 level in the presence of TSH elevation above the upper limit of the normal reference range and < 10.0mIU/L. Thyroid autoantibodies including thyroid peroxidase antibody (TPO-Ab), thyroglobulin antibody (Tg-Ab) and TSH-receptor antibody (TR-Ab), are examined as well, with values of > 34IU/ml, > 115IU/ml and > 1.75IU/L considered positive, respectively.

Diagnosis of fulminant type 1 diabetes mellitus (FT1DM) was established based on published literatures [13, 14]and included all of the following: (1) acute onset of diabetic ketosis or ketoacidosis after the occurrence of hyperglycemic symptoms (including polyuria, polydipsia and thirst) with a duration around one week; (2) high concentrations of plasma glucose (> 16.0mmol/L) with either normal or mildly elevated glycated hemoglobin (HbA1c) < 8.5% at initial presentation; and (3) fasting serum C - peptide level < 0.3ng/ml (reference interval 1.1-4.4ng/ml) and < 0.5ng/ml after meal load, indicating severely impaired pancreatic β-cell secretory capacity.

### Severity classification

The severity of each patient was evaluated based on Common Terminology Criteria for Adverse Events (CTCAE version 5.0). Endocrine-irAEs were graded from asymptomatic (Grade 1) to fatal (Grade 5), with symptoms ranging from mild (Grade 1) to most intense (Grade 3). The severity of IAD was graded based on the criteria of hypopituitarism. Fatigue is a feeling of general weakness with a pronounced inability to summon sufficient energy to accomplish daily activities, grading from relieving by rest (Grade 1) to limiting self-care activity (Grade 3). Nausea is characterized by a queasy sensation and/or the urge to vomit, varying from loss of appetite (Grade 1) to inadequate oral intake (Grade 3). Headache characterized by a sensation of marked discomfort in various parts of the head, not confined to the area of distribution of any nerve.

### Statistical analysis

SPSS 26.0 was used for data analysis. Continuous data that conformed to normal distribution were expressed as mean ± standard deviation (SD), and those that did not conform to normal distribution were expressed as median quartile (P25, P75) and compared by Mann-Whitney U-test. Categorical data were expressed as n/ total n or n/ total n (%) and compared between groups using the Fisher’s exact test. *P* value < 0.05 was considered statistically significant.

## Results

### General information of patients with IAD induced by ICI

**Fig. 1:**
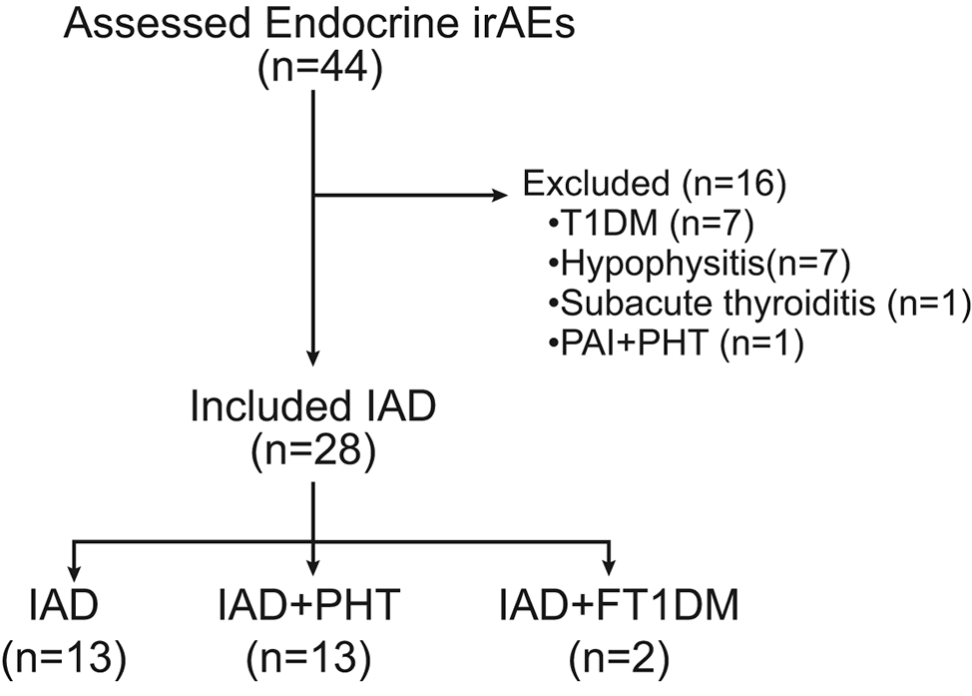
**Flowchart of enrolled patients.** n, number; irAEs, immune checkpoint inhibitors related adverse events; IAD, isolated adrenocorticotropic hormone deficiency; PHT, primary hypothyroidism; FT1DM, fulminant type 1 diabetes mellitus; T1DM, type 1 diabetes mellitus; PAI, primary adrenal insufficiency

In general, a total of 44 patients with ICI-induced endocrinopathies were admitted to our department from January 2019 to August 2022. Among these patients, 28 were diagnosed with IAD. The rest 16 patients were diagnosed with subacute thyroiditis (Grade 1), hypophysitis (all involved 2 anterior pituitary axes, including 3 patients with secondary adrenal insufficiency and secondary hypothyroidism, and 4 patients with secondary adrenal insufficiency and hypogonadotropic hypogonadism), or type 1 diabetes (Fig. 1). Of these 28 patients, 13 (46.4%) were diagnosed only with IAD (Grade 3–4), while the other 13 (46.4%) patients with IAD (Grade 3–4) and PHT (Grade 1–2), the rest two (7.1%) with IAD (Grade 3) and FT1DM (Grade 4). The average ages of patients were 61.2 ± 10.9 years old, with a slight male predominance (16 males vs. 12 females). The tumor types varied (Table 1). The median time to develop IAD was 24 (18–39) weeks, and the earliest was 4 weeks (after 2 cycles). No eosinophilia was observed in patients with IAD in this cohort. Pituitary MRI was performed in 14 patients and all results were normal. Patients who received chemotherapy had ceased this therapy at least three months before hormone testing.

**Table 1:** General information of patients with IAD.

	IAD (n = 13)	IAD + PHT (n = 13)	IAD + FT1DM (n = 2)	Total (n = 28)
Gender (male/female)	8/5	7/6	1/1	16/12
Age, years, mean ± SD	63.0 ± 9.5	58.8 ± 11.5	65	61.2 ± 10.9
Occurrence time, weeks, median (IQR)				
IAD	27 (18–40)	24 (21–33)	18.5	24 (18–39)
PHT	-	24 (21–42)	-	
FT1DM	-	-	18.5	
Type of tumor, n/ total n				
Cervical cancer	1/13	1/13	-	2/28
Nasopharyngeal carcinoma	1/13	-	-	1/28
Lung cancer	2/13	2/13	1/2	5/28
Esophageal cancer	1/13	2/13	-	3/28
Gastric carcinoma	-	1/13	-	1/28
Hepatocellular carcinoma	2/13	3/13	-	5/28
Intrahepatic cholangiocarcinoma	3/13	1/13	-	4/28
Renal cell carcinoma	2/13	2/13	-	4/28
Melanoma	1/13	1/13	-	2/28
Breast cancer	-	-	1/2	1/28
ICIs (dosage, weeks), n/ total n				
sintilimab (200 mg, q3w)	3/13	6/13	1/2	10/28
pembrolizumab (200 mg, q3w)	6/13	1/13	-	7/28
toripalimab (240 mg, q3w)	2/13	-	-	2/28
camrelizumab (200 mg, q2w)	2/13	2/13	-	4/28
tislelizumab (240 mg, q2w)	-	1/13	-	1/28
nivolumab (unknown, q2w)	-	1/13	-	1/28
atezolizumab (1200 mg, q3w)	-	-	1/2	1/28
Drug A in clinical trial (unknown, q3w)	-	1/13	-	1/28
Drug B in clinical trial (unknown, q2w)	-	1/13	-	1/28
Other combination therapy, n/ total n				
chemotherapy	2/13	1/13	1/2	4/28
anlotinib	1/13	2/13	-	3/28
lenvatinib	2/13	1/13	-	3/28
apatinib	1/13	-	-	1/28

Abbreviations: n, number; ICI, immune checkpoint inhibitors; IAD, isolated adrenocortipic hormone deficiency; PHT, primary hypothyroidism; FT1DM, fulminant type 1 diabetes mellitus; IQR, interquartile range

### Characteristics of patients with IAD alone

**Fig. 2:**
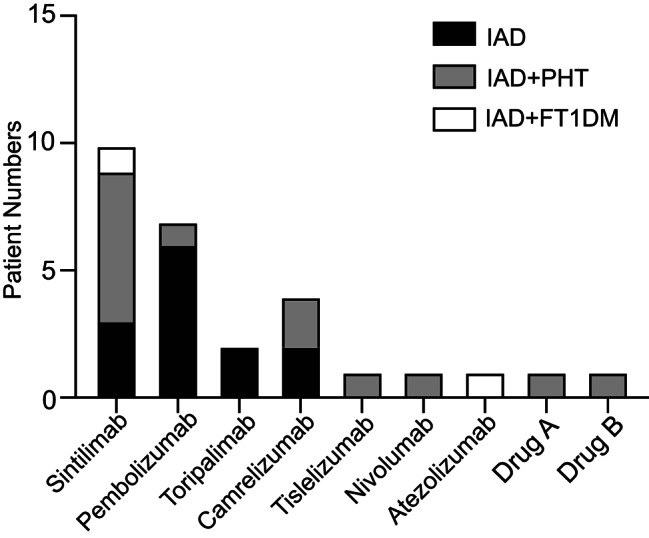
**Numbers of patients with IAD induced by different immune checkpoint inhibitors.** Drug A and drug B are currently undergoing clinical trials. IAD, isolated adrenocorticotropic hormone deficiency; PHT, primary hypothyroidism; FT1DM, fulminant type 1 diabetes mellitus

Among the thirteen patients with IAD, four were treated with sintilimab, four with pembrolizumab, three with camrelizumab, and two with toripalimab (Fig. 2). The main symptoms were fatigue (Grade 2–3) and nausea (Grade 2). Headache (Grade 1) was very mild in only one patient. Only three patients experienced hypotension (Grade 3). Nine patients showed hyponatremia (seven in Grade 4 and two in Grade 3), and blood potassium was all within the normal range. Of six patients who received intravenous hydrocortisone, two experienced agitation (Grade 3) after using 100–200 mg of intravenous steroids. Seven patients were prescribed oral cortisone acetate instead of intravenous corticosteroids due to relatively mild symptoms. The median dose of oral cortisone acetate was 37.5 (37.5, 50.0) mg at discharge (Table 2).

**Table 2:** Clinical characteristics of patients with IAD.

	IAD (n = 13)	IAD + PHT (n = 13)	IAD + FT1DM(n = 2)	*P*
Symptoms, n/total n (%)				
Fatigue	13/13	13/13	2/2	-
Nausea	11/13 (84.6)	8/13 (61.5)	1/2 (50)	0.378^a^
Headache	1/13 (7.7)	2/13 (15.4)	0	1.000^a^
Polydipsia	-	-	2/2	-
Hypotension, n/n tested (%)	3/13(23.1)	1/13(7.7)	0/2	0.593^a^
Hyponatremia, n/n tested (%)	9/13(69.2)	8/13(61.5)	2/2	1.000^a^
Laboratory results (unit, normal range), median (IQR)				
Morning ACTH (pmol/L, 1.5–13.9)	0.46 (0.33, 2.24)	0.40(0.33, 1.25)	1.54	0.511^a^
Morning Cortisol (nmol/L, 133–537)	19.7(7.3, 45.8)	15.5 (5.1, 37.0)	11.47	0.840^a^
TSH (mIU/L, 0.27–4.2)	1.89(1.48, 3.7)	9.92(6.43, 36.8)	1.87	< 0.01^a^
FT4 (pmol/L, 12.0–22.0)	16.3 (14, 17.4)	11.1 (8.2, 15.7)	13.5	< 0.01^a^
Thyroid autoantibodies, n positive/n tested (%)				
TPO-Ab	3/13(23.1)	2/13(15.4)	0/2	-
Tg-Ab	1/13(7.7)	1/13(7.7)	0/2	-
TR-Ab	0/13	0/13	0/2	-
GAD/IAA/ICA, n positive/n tested	-	-	0/2	-
Pituitary MRI, normal n/ n performed	7/7	7/7	-	-
Numbers using intravenous glucocorticoid, n/ total n	6/13	6/13	-	-
Intravenous glucocorticoid (mg), median (IQR)	225(150, 350)	200(50, 250)	0	0.481 ^a^
cortisone acetate at discharge (mg), median (IQR)	37.5 (37.5, 50.0)	37.5(25.0, 37.5)	37.5	0.56^a^
Levothyroxine at discharge (µg), median (IQR)	-	25(12.5, 75.0)	-	-

Notes: a, comparison between patients with IAD and IAD + HT.

Abbreviations: n, number; IAD, isolated adrenocorticotrophic hormone deficiency; PHT, primary hypothyroidism; FT1DM, fulminant type 1 diabetes mellitus; IQR, interquartile range; ACTH, adrenocorticotrophic hormone; TSH, thyroid stimulating hormone; FT4, free thyroxine; TPO-Ab, thyroid peroxidase antibody; Tg-Ab, thyroglobulin antibody; TR-Ab, TSH-receptor antibody; GAD, anti-glutamic acid decarboxylase; IAA, insulin autoantibody; ICA, Islet cell antibody; MRI, Magnetic Resonance Image

### Characteristics of patients with IAD and PHT

Among the 13 patients with IAD and PHT, 5 were treated with sintilimab, and 3 with pembrolizumab, 3 with pembrolizumab, nivolumab, and tislelizumab respectively. Two patients were on drug A and B both of which were still in clinical trials (drug A, an anti-PD-1 and anti-CTLA4 bispecific antibody; drug B, an anti-PD-1 antibody) (Fig. 2). Five patients showed PHT first, followed by IAD with interval of 4–20 weeks, while six were diagnosed with both conditions at the same time. Only 1 patient who used sintilimab developed PHT subsequent to IAD, with an interval of 21 weeks. Fatigue (Grade 2–3), nausea (Grade 2) and headache (Grade 1) were also reported in this group. Eight patients experienced hyponatremia (five cases in Grade 4, and three in Grade 3). Only two patients tested positive for thyroid autoantibodies (one patient with positive TPO-Ab and Tg-Ab, the other one with only positive TPO-Ab). Six patients were administered intravenous corticosteroids, with a median dose of 200 (50–250) mg. One of them experienced agitation (Grade 3), which was relieved after stopping glucocorticoid temporarily. Five patients with subclinical hypothyroidism did not take levothyroxine. The median dose of levothyroxine taken at discharge was 25 (12.5, 75.0) µg (Table 2). No significances were indicated in levels of morning ACTH, cortisol and usage of glucocorticoids between patients with IAD and those with IAD and PHT.

### Characteristics of patients with IAD and FT1DM

Two patients taking atezolizumab and toripalimab respectively, developed FT1DM and IAD at 16 weeks and 21 weeks respectively (Fig. 2). It is noteworthy that both patients reported mild elevation in blood glucose levels, and then progressed to diabetic ketoacidosis (DKA) within 4 weeks. IAD was diagnosed at the same time. They experienced fatigue (Grade 3), nausea (Grade 2), vomiting, thirst and polydipsia. HbA1c levels were 8.4% and 8.0%, respectively. All diabetic autoantibodies, including anti-GAD, insulin autoantibody, and islet cell antibody, were negative in both patients. Fasting insulin and C-peptide were also below the lower limit of detection. Intravenous insulin and fluid resuscitation were started immediately. Both patients were treated with oral cortisol instead of intravenous corticosteroids. They received standard insulin injection regimen with daily doses of 22 and 33 units respectively (Table 2).

### Follow-up

All patients continued glucocorticoids regimen in the following 3–6 months. Only one patient with poorly differentiated neck adenocarcinoma continued sintilimab. The other 27 patients discontinued ICI treatment in the follow-up. Unfortunately, one patient died of tumor metastasis, while the rest continued to receive other treatments.

## Discussion

In this study, we retrospectively analyzed 28 patients with IAD caused by anti-PD-1/PD-L1. Over half of the patients (53.5%) had an additional endocrinopathy. PHT was more frequently observed than FT1DM in patients with IAD, but the latter presented with more severe symptoms. Fatigue and nausea were the primary complaints. Hyponatremia (67.8%) was a common laboratory finding. All patients continued oral hydrocortisone regimen during 3-6-month follow-up. To our knowledge, this is the first study to provide a comprehensive overview of IAD patients under ICI therapy.

In our study, IAD was mainly caused by anti-PD1/PD-L1, with the exception of one case induced by a bispecific antibody (drug A in clinical trials). IAD is more common in anti-PD1 therapy, as opposed to whole hypophysitis caused by anti-CTLA4 [7, 15]. There is no consensus on whether IAD by ICIs can be classified as hypophysitis, whereas ACTH is more commonly affected than thyrotrophs and gonadotrophs in anti-CTLA4-induced hypophysitis [16]. The exact mechanism of IAD by ICI is yet to be determined. However, some studies suggested that it may be associated with the induction of anti-pituitary antibodies by ICI [17, 18]. Additionally, a special phenotype of human leukocyte antigen is also a possible predisposing factor for pituitary irAE [18, 19].

Patients in our research have some similarities with those in prior studies. It has been observed that ICI-IAD usually occurs several months to over a year after initiating ICI treatment [20]. Additionally, men at an elder age are more likely to be affected [16]. Hyponatremia tends to be one of the most common presenting findings [7]. It is important to note that patients with IAD may also experience nonspecific and sometimes mild symptoms that are commonly seen in malignancy, such as fatigue, nausea, and headache. Therefore, when encountering patients with suspected endocrine irAE, it is advisable to inquire whether their symptoms have suddenly worsened or persisted in the absence of tumor progression. Regular assessment of hormones and serum electrolytes seems is crucial in immunotherapy and can provide a more reliable way of differentiating ICI-related endocrinopathies from other conditions, as compared to relying only on symptoms and physical examination.

In previous reports, abnormalities in pituitary MRI are present in 81% of cases due to anti-CTLA-4, while in 18% of patients with hypophysitis caused by anti-PD-1/PD-L1, the initial enlargement of pituitary tends to resolve within weeks [7, 20]. As seen in our patients, no abnormalities in pituitary MRI were indicated, which is in agreement with the previous study [21]. Nevertheless, a normal MRI scan does not exclude hypophysitis, and imaging is still required to diagnose hypophysitis and to exclude other sources of pituitary failure, such as metastatic disease [22]. Although high-dose glucocorticoid is recommended in case of suspected acute adrenal crisis (usually 100 mg hydrocortisone intravenously followed by 50 mg every 6 h and fluid resuscitation) [10], we noticed that 3 patients in our study developed hyperactivity after 200–300 mg hydrocortisone intravenously, and recovered after temporary discontinuation of glucocorticoid. Thus, it is important to be aware of any alterations in patients’ symptoms in hormone replacement therapy.

Thyroid dysfunction is common in anti-PD1 induced irAEs [7, 23]. Previous studies have reported that approximately 38% of patients undergoing anti-PD-1 treatment may experience thyroid dysfunction, including subclinical disease [21, 24]. Additionally, up to 50–70% of them may develop thyroid antibodies [25, 26]. However, our research found that most patients who developed hypothyroidism were tested negative thyroid antibodies. There are several possible reasons for the low negativity rate of thyroid antibody in our study. Firstly, although the elevated levels of Tg-Ab and TPO-Ab have been identified as a potential risk factor for future development of ICI associated thyroid dysfunction [26], the exact role of thyroid antibodies in the pathogenesis of ICI-PHT remains unclear. It is possible for autoimmune thyroiditis to be present even in the absence of detectable thyroid antibodies, which may lead to higher serum TSH levels that in turn might be the basis of the increased incidence of hypothyroidism in such patients [27]. Some patients who develop ICI-related thyroid dysfunction without elevated titers of thyroid antibodies at the time of abnormal thyroxine, suggesting TPO-Ab and Tg-Ab are not necessary for the development of ICI-related thyroid dysfunction [2]. Secondly, although the development of positive thyroid antibodies after the initiation of immunotherapy is associated with higher risks for overt thyroid dysfunction [25], thyroid dysfunction usually arises before the shift of thyroid antibody from negative to positive [28]. In this study, some patients exhibited subclinical hypothyroidism with minor deviations in thyroid hormone levels, and insufficient elevation of thyroid antibodies. As a result, the majority of the patients remained in a negative state. Finally, it is worth noting that while anti-PD1 drugs can be categorized under one class, there may still be variations among different drugs. In our research, sintilimab, an anti-PD1 approved in China, was applied in most patients. Although thyroid dysfunction is the most commonly reported irAE of sintilimab [29], there is limited information available on the effect of thyroid antibodies by sintilimab. Therefore, it is reasonable to speculate that the low positive rate of thyroid antibodies in this study may be related to the use of different ICIs.

In our study, five patients (5/13, 38.5%) were presented with subclinical hypothyroidism, therefore did not take LT4. This finding was consistent with previous studies in which the thyroid alteration in most patients with hypothyroidism was mild or subclinical even with extreme laboratory values, therefore did not require immediate pharmacological intervention [4, 26]. For these patients, treatment for hypothyroidism can begin when needed [24]. Therefore, IAD is the prominent and pressing concern for these patients, rather than hypothyroidism. Despite the fact that some patients display elevated hypothyroidism markers, the concomitant presence of IAD can complicate the identification of typical hypothyroidism symptoms such as fatigue, anorexia, and edema. When dealing with cases of concurrent hypothyroidism and IAD, it is critical to administer glucocorticoid prior to thyroxine to prevent adrenal crisis. Our study revealed that some patients developed IAD following PHT, reminding physicians to be aware of the potential risk of this condition during immunotherapy.

In our research, two patients developed IAD and FT1DM after being treated with atezolizumab and sintilimab respectively. The incidence of ICI-T1DM is low, ranging from 0.2 to 1.4% [30], mainly due to anti-PD-1/PD-L1 [31]. The immune etiology of FT1DM and IAD may be more evident when they co-occur. Destruction of β-cells is more severe in ICI-T1DM in comparison with classic T1DM [32]. In contrast, only 50% of patients with ICI-T1DM have a relevant autoantibody, with anti-GAD being the majority [33]. DKA at diagnosis is very frequent, up to 70% [7]. In our study, the C-peptide level was too low to be detected, suggesting severe impairment of islet β cells. ICI-FT1DM combined with IAD is rare and can be fatal, but the treatment in this condition could be conflicting. On one hand, both hormones must be replaced instantly due to profound lack of insulin and glucocorticoid. On the other hand, glucocorticoids will increase serum glucose, potentially worsening hyperglycemia and DKA. In our study, we applied oral glucocorticoids to these patients and obtained remission. This approach may be an optimal alternative in this condition.

Previous studies have largely focused on the incidence of single endocrine irAEs. However, people with one autoimmune disease are more likely to develop a second autoimmune disease [34]. Growing evidence suggests that polyendocrinopathy is common following ICI treatment, similar to autoimmune endocrine diseases in general. ICI-induced polyendocrinopathy typically manifests as a combination of thyroid disorder and other endocrinopathy, such as hypophysitis, T1DM, or primary adrenal insufficiency, known as autoimmune polyendocrine syndrome (APS) [35]. It is assumed that ICI-induced APS shares a similar occurrence rate with the classic type [7]. It is possible to classify a combination of IAD and other endocrinopathies as a new type of APS. From this point, the endocrine irAEs can provide new insights into the pathogenesis of autoimmune endocrine disorders [36].

Our study has several limitations that should be taken into consideration. Firstly, it was a retrospective observational study with a relatively small sample size, thus further research with a larger sample size is needed to gain a more comprehensive understanding of IAD. Secondly, only patients admitted to the Department of Endocrinology were included, indicating those with mild symptoms were not considered. Thirdly, the follow-up period was short. Therefore, we were unable to determine the relationship between irAEs and treatment effect. Finally, despite similar mechanisms and potential adverse reactions, the prevalence of endocrine irAEs largely depends on the utilization of different ICIs. For instance, some drugs in this study, such as sintilimab, toripalimab, camrelizumab, tislelizumab, are not approved for certain use outside China, thus restricting some of the findings in our research.

## Conclusion

In conclusion, over half of ICI-related IAD (53.5%) is co-occurred with other types of endocrine irAEs. Primary hypothyroidism is the most common endocrine irAE accompanied with IAD, while co-occurrence of FT1DM and IAD is rare but life-threatening. Fatigue and nausea are the chief complaints. Hyponatremia (67.8%) is common in laboratory findings. This damage could happen at any stage of ICI treatment. Due to lack of predictable biomarkers for IAD and its nonspecific symptoms in patients with cancer, regular assessment of morning cortisol and ACTH is essential and reliable. It is necessary to pay close attention to the patient’s endocrine system irAEs for long term, even after ending ICI treatment.

## Data Availability

The datasets used and/or analyzed during the current study are available from the corresponding author on reasonable request.

## Abbreviations

ICI: Immune checkpoint inhibitor
PD-1: Programmed cell death receptor-1
PD-L1: Programmed cell death ligand-1
CTLA-4: Cytotoxic-T cell lymphocyte antigen-4
irAE: Immune related adverse effect
IAD: Isolated adrenocorticotropic hormone deficiency
PHT: Primary hypothyroidism
FT1DM: Fulminant type 1 diabetes mellitus
T1DM: Type 1 diabetes mellitus
MRI: Magnetic resonance imaging
ACTH: Adrenocorticotropic hormone
TSH: Thyroid stimulating hormone
TPO-Ab: Thyroid peroxidase antibody
Tg-Ab: Thyroglobulin antibody
TR-Ab: TSH-receptor antibody
anti-GAD: Anti-glutamic acid decarboxylase
IAA: Insulin autoantibody
ICA: Islet cell antibody
APS: Autoimmune polyendocrine syndrome
